# A Comparative Study of the Safety and Efficacy of Intrapleural Fibrinolysis With Streptokinase and Urokinase in the Management of Loculated Pleural Effusions

**DOI:** 10.7759/cureus.26271

**Published:** 2022-06-24

**Authors:** Khushboo Saxena, V. Nagarjuna Maturu

**Affiliations:** 1 Respiratory Medicine, Gandhi Medical College, Bhopal, IND; 2 Department of Interventional and Clinical Pulmonary Medicine, Yashoda superspeciality Hospital, Hyderabad, IND

**Keywords:** tubercular effusions, syn-pneumonic effusion, loculated pleural effusions, urokinase, streptokinase, intrapleural fibrinolysis

## Abstract

Background

Intrapleural fibrinolytic therapy (IPFT) with streptokinase (STK), urokinase (UK), and alteplase remains a common practice for managing loculated pleural effusions (LPEs). However, very limited data are available on the comparative efficacy of these agents.

Methodology

We compared the efficacy and safety of intrapleural streptokinase (n = 28) and urokinase (n = 38) in 66 patients with loculated effusions. IPFT was initiated if effusion remained undrained despite the placement of intercostal chest drainage or pigtail catheter. The dose of STK and UK were 250,000 IU twice daily and 100,000 IU once daily, respectively. The volume of fluid drained after IPFT, radiologic response, clinical response, and adverse events were compared between the two groups.

Results

The mean volume of fluid drained post-IPFT was 1,379 mL in the STK arm and 1,110 mL in the UK arm (p = 0.251). Of the 66 patients, 53 (80.3%) had good clinical response, and 28 (43.7%) had >75% resolution of effusion on chest radiographs. The clinical (79% vs. 82%; p = 0.765) and radiologic response rates (39.3% vs. 44.6%; p = 0.568) were similar in both STK and UK arms. Pain was the most common adverse event in both groups. Significantly more patients in the STK arm developed fever (14% vs. 0%, p = 0.030). Treatment-limiting adverse events occurred in five patients.

Conclusions

IPFT is a safe and effective method for managing patients with LPEs. Although the clinical and radiologic response rates were similar with STK and UK, the latter may be the preferred choice because of its better safety profile and ease of administration (once-daily dose).

## Introduction

The organization of pleural fluid within the pleural cavity gives rise to loculated pleural effusions (LPEs). Loculation is the result of intense intrapleural inflammation and organization and is most commonly due to infections, malignancies, and hemothorax. If left untreated, LPEs lead to pleural thickening, fibrothorax, trapped lung, and can compromise lung function. The three common causes of LPE are pneumonia, tuberculosis, and malignancy. Intrapleural fibrinolytic therapy (IPFT) has been shown to be beneficial in the management of LPE of any etiology [1-4].

Empyema and parapneumonic effusions develop in approximately 36-66% of patients hospitalized with bacterial pneumonia [5]. Loculations develop early if left undrained, and this leads to a prolonged hospital stay and increased morbidity and mortality. In developing countries like India, tuberculosis remains an important cause of LPE. Fibrothorax, a late sequela of untreated loculated tubercular effusion, is still common. Another cause of LPE is malignancy. Pleural fluid loculations or trapped lungs frequently render patients with symptomatic malignant pleural effusions (MPEs) unsuitable for pleurodesis.

First-line management of LPE usually comprises drainage through a chest tube and treatment of the disease process. However, it fails in approximately one-third of patients who then require additional interventions. For almost 70 years, IPFT has been a part of the therapeutic armamentarium to expedite pleural drainage in patients with empyema and complicated parapneumonic pleural effusions [6]. Nevertheless, numerous controversial points remain, including the drug to be used, the role of the combination of fibrinolytic with other enzymes such as deoxyribonuclease (DNAse), and the optimal dose, dosing interval, and duration of IPFT [7].

Early, small, but well-conducted randomized placebo-controlled trials of the fibrinolytic urokinase (UK) and streptokinase (STK) suggested a benefit to surrogate outcomes in adults, such as chest tube fluid output, favorable radiological change, and decreased surgical referral rates [6,8-10]. However, the two large and adequately powered randomized trials, the Multicenter Intrapleural Sepsis Trial, MIST1 and MIST2, demonstrated a lack of utility in using IPFT alone compared with placebo in adults [11,12]. Whether the results of these trials are generally applicable remains an open question. Consequently, the administration of IPFT or decisions to use it instead of early surgery is based on local experience and availability. Despite negative results from MIST trials, it is a common clinical practice in the developing world to use STK and UK monotherapy for draining LPEs [13,14].

Of the three fibrinolytic agents available for intrapleural fibrinolysis, namely, STK, UK, and alteplase, the superiority of alteplase in combination with DNAse has been documented [12,15]. However, in developing nations, the use of alteplase is restricted due to its high cost. Although many studies have evaluated each fibrinolytic agent against a placebo, there are only two studies to date that have compared the efficacy of these fibrinolytic agents [16,17]. In our study, we aim to assess and compare the efficacy and safety of UK and STK monotherapy as fibrinolytic agents for managing LPEs.

## Materials and methods

This prospective, observational study was conducted at Yashoda Superspeciality Hospital, Somajiguda, Hyderabad after obtaining approval from the Institutional Ethical Committee of Yashoda Academy of Medical Education and Research (ECR/49/Inst/AP/2013/RR-16).

All consecutive patients admitted with LPE during a two-year period (June 2016 to May 2018) were included. Patients aged 18 years and above were eligible for the study if they had LPE confirmed by chest ultrasonography or a computed tomogram of the chest. All such patients underwent placement of a pigtail catheter or an intercostal thoracostomy tube. After placement of an intercostal drain or a pigtail catheter, the free-flowing fluid was drained. Once the drain fluid output stopped, patients were reassessed by a chest radiograph and a bedside pleural ultrasonography. Those who had residual loculated pleural collection which was not drained by the intercostal drain were included in this study for intrapleural fibrinolysis. After obtaining written informed consent, patients received either intrapleural UK or STK, as per physician discretion. Patients who had a history of allergic reaction to STK and those with alveolo-pleural/ broncho-pleural fistulae were excluded from the study. The cause of the effusion was determined based on the clinicoradiological picture, pleural fluid analysis, and pleural biopsy report (when performed).

Intrapleural fibrinolysis procedure

For STK, 250,000 IU STK diluted in 20 mL normal saline (NS) was instilled intrapleural every 12 hours. This was followed by a 20 mL NS flush, and the tube was clamped for two hours after the instillation. For UK, 100,000 IU UK diluted in 20 mL NS was instilled intrapleural every 24 hours. This was followed by a 20 mL NS flush, and the tube was clamped for two hours after instillation.

Intercostal drain output, visual analog scale (VAS) pain score, and adverse effects were monitored after each dose. Chest radiograph was repeated after every three doses or if the drain output was reduced to less than 50 mL after two consecutive doses. Instillation of intrapleural agents was stopped when the drain output was less than 50 mL per day, or after complete resolution of pleural effusion, as seen on an ultrasound chest. Radiologic resolution of the effusion on chest X-ray (CXR) was assessed by a radiologist and a pulmonologist independently. The resolution was categorized as follows: (a) complete response (>90% resolution of the effusion on the CXR); (b) near-complete response (75-90% resolution of the effusion on the CXR); (c) partial response (25-75% resolution of the effusion on the CXR); (d) no response (less than 25% resolution of the effusion on the CXR). Any differences in opinion between the two were settled after discussion.

Patients who continued to remain symptomatic and with no response on CXR were subjected to alternative interventions, as deemed necessary.

Statistical analysis

All measured values are reported as mean (standard deviation) for continuous variables and as frequencies and percentages for categorical variables. The Student’s t-test was used to assess differences between means, and for categorical variables, the chi-square test/Fisher’s exact test was applied. Results were considered significant at a p-value of <0.05. All analyses were performed using the Statistical Package for the Social Sciences (SPSS) version 18.0 (SPSS Inc, Chicago, IL, USA) and MedCalc version 9.1 (MedCalc Software, Mariakerke, Belgium).

## Results

A total of 68 patients were recruited during the study period of two years (June 2016 to May 2018). Two patients died due to progressive respiratory failure prior to initiation of IPFT. Others (n = 66) received IPFT with either STK (n = 28) or UK (n = 38). The mean age of the study population was 43.4 ± 17.1 years. There were 45 (68%) males and 21 (32%) females. The most common diagnosis was syn-pneumonic effusion (24/66; 36.4%) and tubercular pleural effusion (24/66; 36.4%), followed by malignant effusion (12/66; 18.2%) (Table 1). The common presenting symptoms were fever (45/66; 68.2%), cough (51/66; 77.3%), dyspnea (59/66; 89.4%), loss of appetite (38/66; 57.6%), loss of weight (36/66; 54.5%), and chest pain (24/66; 36.4%). The mean duration of symptoms was 26.7 ± 28.2 days for the STK group and 25.5 ± 29.2 days for the UK group (Table 1).

**Table 1:** Clinico-demographic parameters and pleural fluid characteristics of the study population. * Values expressed as x ± y = mean ± standard deviation; ^#^ Values expressed as N (percentage).

	Streptokinase (n = 28)	Urokinase (n = 38)	P-value
Age (years)*	47.6 ± 15.7	40.1 ± 19.9	0.081
Sex (Male/Female)	19/9	28/10	0.961
Comorbidities			
Diabetes mellitus#	8 (28)	6 (16)	0.209
Systemic hypertension#	10 (36)	13 (34)	0.899
Chronic kidney disease#	3 (11)	3 (8)	0.693
Clinical symptoms			
Fever#	19 (68)	26 (68)	0.691
Cough#	22 (78)	29 (76)	0.829
Dyspnea#	21 (75)	28 (74)	0.904
Chest pain#	5 (18)	19 (50)	0.007
Loss of appetite#	15 (54)	23 (60)	0.572
Loss of weight#	13 (46)	23 (60)	0.256
Side of effusion (right/left)	14/14	19/19	1.000
Type of drain			
Pigtail#	13 (46)	19 (50)	0.774
Intercostal chest drainage tube#	15 (54)	19 (50)	
Pleural fluid characteristics			
Total leucocyte count (cells/mm^3^)*	2,804 ± 4,186.0	2,621 ± 4,669.9	0.886
Polymorphs (%)*	39.5 ± 35.7	27.2 ± 34.9	0.17
Lymphocytes (%)*	51.9 ± 33.6	67.2 ± 34.6	0.18
Protein (g/dL)*	3.9 ± 1.7	4.5 ± 1.48	0.176
Sugar (mg/dL)*	88.1 ± 45.4	79.8 ± 52.1	0.534
Adenosine deaminase (IU/L)*	59.1 ± 51.7	53.1 ± 82. 6	0.757
Diagnosis			
Syn-pneumonic effusion#	11 (39.2)	13 (34.2)	
Tubercular effusion#	10 ( 35.7)	14 (36.8)	
Malignant effusion#	4 (14.3)	8 (21.0)	
Hemothorax#	2 (7.1)	0	
Others**#	1 (3.5)	3 (7.8)	

**Figure 1:**
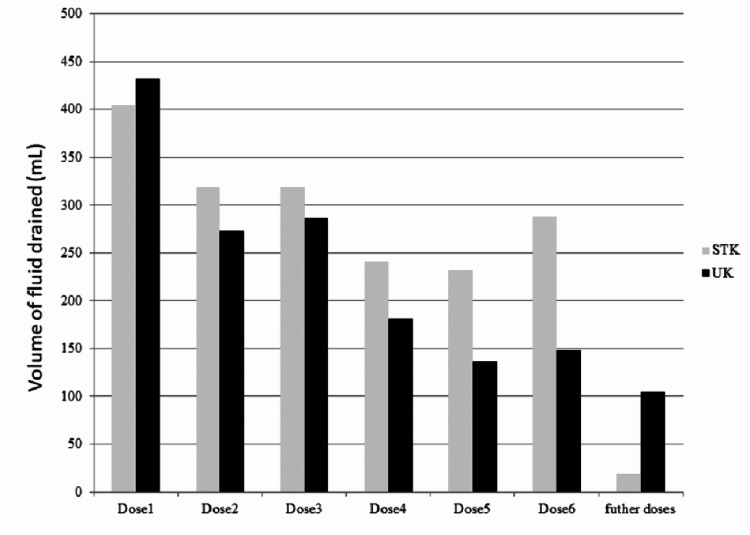
Mean volume of fluid drained (in mL) after each dose of STK and UK. STK: streptokinase; UK: urokinase

Average number of doses instilled in the STK and UK groups were 4.3 ± 1.4 and 4.00 ± 2.7, respectively. The mean number of days for which they received IPFT was 3.3 ± 1.8 and 4.2 ± 2.7, respectively (Table 2). The average volume of fluid drained after STK instillation was 1,379.2 ± 771.9 mL and after UK was 1,109.9 ± 1,036.9 mL (Figure 1). The difference was not significant between the two groups (p = 0.251). Pre-STK radiographs were not available for two patients. Hence, they were not included in the radiologic response analysis. After IPFT, 28 (43.75%) patients had >75 % radiological resolution, and 12 (18%) patients had partial response. The radiologic response was similar between the two groups (p = 0.568). The clinical response was assessed subjectively on the basis of relief of symptoms and drain output. Of the 66 patients included, 53 (80.30%) improved clinically after IPFT. The clinical response rate was 78.6% in the STK arm and 81.6% in the UK arm (p = 0.765). Of the 13 patients, who did not respond, eight required additional procedures, including ultrasound-guided aspiration from residual loculi (n = 3), thoracoscopic adhesiolysis (n =2), surgical decortication (n = 2), and repositioning of the pigtail catheter (n = 1) (Table 2).

**Table 2:** Treatment outcomes of intrapleural fibrinolytic therapy. * Values expressed as x ± y = mean ± standard deviation; ^#^ Values expressed as N (percentage); ** Additional doses administered after the sixth dose. IPFT: intrapleural fibrinolytic therapy

	Streptokinase (n = 28)	Urokinase (n = 38)	P-value
Number of IPFT doses administered	4.3 ± 1.4	4.00 ± 2.7	0.617
Number of days	3.3 ± 1.8	4.2 ± 2.7	0.139
Volume drained pre-fibrinolysis (mL)	900.4 ± 843.4	1,150.7 ± 1,326.9	0.385
Volume after each dose (mL)			
Dose 1	404.3 ± 263.3	432.7 ± 455.8	0.769
Dose 2	318.2 ± 255.8	272.6 ± 273.0	0.494
Dose 3	318.8 ± 214.2	286.8 ± 235.0	0.608
Dose 4	241.2 ± 147.9	181.5 ± 136.3	0.230
Dose 5	231.3 ± 185.2	136.4 ± 143.3	0.170
Dose 6	287.5 ± 240.2	148.3 ± 140.8	0.232
Further doses*	18.4 ± 60.6	104.3 ± 339.1	0.283
Total volume drained (mL)	1,379.2 ± 771.9	1,109.9 ± 1,036.9	0.251
Radiological resolution			
Complete	4 (14.3%)	6 (15.7%)	0.568
Near-complete	7 (25.0%)	11 (28.9%)	
Partial	7 (25.0%)	5 (13.2%)	
No response	8 (28.6%)	16 (42.1%)	
Clinical response	22 (78.6%)	31 (81.6%)	0.765
Need for additional procedure	3 (11%)	5 (13%)	0.643

Pain was the most common adverse effect observed after fibrinolysis. Pain could not be assessed in four patients as they were on a ventilator for underlying disease. Of the 62 patients, 57 (91.1%) experienced pain after IPFT. Severe pain (VAS > 5) was experienced by 12 (19.1%) patients. Overall, 33.33% of patients in the STK group and 15.15% of patients in the UK group developed severe pain (p = 0.217). The mean pain VAS score was also similar between the two arms. Significantly more patients in the STK arm developed fever after fibrinolysis (4/28 vs. 0/38; p = 0.030). One patient developed an allergic reaction to STK, and two patients developed hemorrhagic conversion of pleural effusion. Treatment-limiting adverse events occurred in five patients. These included severe pain (n = 1), hemorrhagic conversion (n = 2), allergic reaction (n = 1), and hypoxia (n = 1) (Table 3).

**Table 3:** Adverse events associated with intrapleural fibrinolysis therapy. * Values expressed as x ± y = mean ± standard deviation; ^#^ Values expressed as n (percentage). VAS: visual analog scale

Adverse event	Streptokinase (n = 28)	Urokinase (n = 38)	P-value
Pain (mean VAS score)*	3.3 ± 2.6	3.0 ± 2.0	0.673
Fever#	4 (14)	0	0.030
Allergic reaction#	1 (3)	0	0.424
Hemorrhage#	0	2 (5)	0.504
Treatment-limiting adverse events	3 (10)	2 (5)	0.643

## Discussion

This study reconfirms the efficacy and safety of IPFT monotherapy in patients with LPEs. Clinical response rates of 80% were achieved with both STK and UK. Fibrinolysis was well tolerated in the majority, and treatment-limiting adverse events occurred in only five patients.

Intrapleural fibrinolytics have been used in the management of LPEs for nearly 70 years. The first use of STK was described in 1951 by Tillett and Sherry in their case series of 25 patients [18]. Multiple small observational studies published since then have shown beneficial effects of intrapleural STK [8,19,20]. However, the MIST1 randomized trial did not show any benefit of intrapleural STK over placebo in terms of mortality, surgical conversion rates, radiographic outcomes, or length of hospital stay. Serious adverse events (chest pain, fever, or allergy) were more common with STK [11]. This dampened the enthusiasm for use of intrapleural STK. The first use of intrapleural UK was demonstrated in 1989 by Moulton et al. [21]. There are limited data on the comparison of the safety and efficacy of STK and UK [16]. To the best of our knowledge, ours is the second study comparing the efficacy and safety of STK and UK.

In our study, the mean volume of fluid drained in the STK and UK groups was 1,379.23 ± 771.9 mL and 1,109.87 ± 1,036.9, respectively (p = 0.251). Our results are concordant with the study conducted by Bouros et al. [16], where the total volume of fluid drained after treatment was 1,596 ± 68 mL for the STK group and 1,510 ± 55 mL for the UK group (p > 0.05). The number of doses required for IPFT (4.3 vs. 4.0; p = 0.617) and the number of days for IPFT were similar between the two study arms.

In our study, 80.3% of the patients had good clinical improvement, which was comparable in the STK and UK arms (78.6% vs. 81.6%). Earlier observational studies on IPFT also showed clinical improvement rates ranging between 66% and 96% [22-24]. Radiologic response rates of our study (>75% improvement in 42.2% of the study population) were lower compared to earlier studies on IPFT [22,25]. Radiologic response rates were similar with STK and UK (p = 0.568). In the study by Bouros et al. [16], 80% of patients had more than two-thirds improvement on chest radiographs. Similarly, in the MIST1 trial [11], 87% of patients had >75% radiologic improvement. The lower radiologic response rates in our study could be partially explained by the reason that we included malignant effusions and tubercular effusions unlike earlier studies which included only parapneumonic effusions. In such patients, the presence of pleural thickening would lead to residual radiologic opacities despite optimal fluid drainage, which would lead to a lower radiologic response rate.

Pain was the most common adverse effect noted in patients undergoing IPFT. More patients in the STK group developed fever after fibrinolysis (14% vs. 0%; p = 0.030). Only one patient developed an allergic reaction after STK administration. None of the patients had life-threatening adverse reactions, and treatment-limiting adverse reactions occurred in only 7.5% of patients. In earlier studies also, STK was more often associated with fever, rash, and allergic reactions [26].

Our study has few limitations. First, it lacked randomization. Second, we compared radiological response using CXR and not chest ultrasonography. Chest radiographs do not differentiate between pleural effusion and pleural thickening, the latter can be present in long-standing effusions such as tuberculous and malignant effusions. This could partially explain the discordance between the radiologic and clinical response rates (43.7% vs. 80.3%). Lastly, it is a single-center study. The results of our study need to be replicated from other centers.

Considering the good clinical response in our study (81%) and an acceptable safety profile, IPFT monotherapy can be considered for the management of LPEs. Although both STK and UK are equally efficacious, UK may be preferred over STK because of lesser febrile reactions and easy (once daily) dose regimen.

## Conclusions

IPFT with STK or UK monotherapy for LPEs has a clinical response rate of 80.3%. IPFT was well tolerated, with treatment-limiting adverse events occurring in only 7.5% of the study population. The clinical and radiologic response rates were similar with STK and UK. In resource-limited settings, we recommend the use of either STK or UK for the management of LPEs. We suggest the use of UK over STK because of fewer febrile reactions with UK and ease of once-daily administration.
